# Advancing system of systems engineering using intangible value logic measurements from intellectual capital thinking approach

**DOI:** 10.1016/j.heliyon.2024.e39814

**Published:** 2024-10-28

**Authors:** Ashraf Zaghwan, Yousef Amer, Mahmoud Efatmaneshnik, Indra Gunawan

**Affiliations:** a Faculty of Sciences, Engineering, and Technology, Adelaide, Australia; b Faculty of Science, Technology, Engineering and Maths, University of South Australia, Adelaide, Australia; c Faculty of Arts, Business, Law and Economics, The University of Adelaide, Adelaide, Australia

**Keywords:** Sustainability, Intellectual capital thinking, Energy loss, Intangible assets, Value shop logic, Value network logic and value chain logic

## Abstract

This study explores the integration of intangible value logic measurements derived from the Intellectual Capital Thinking (IC_*T*_) approach to advance System of Systems Engineering (SoSE). Focusing on demand-side electricity management, the research illuminates the challenges and opportunities in capturing intangible value creation within engineering systems. By delving into human behaviour-driven demand intricacies and recognising intangible resources among stakeholders, the study unveils the potential for innovation in demand management strategies. Through the analysis of three interacting value logics—shop, chain, and network—the research elucidates their impact on system performance. Addressing the practical application gap, the study introduces ICT as a strategic lens to enhance performance through non-monetary values. Qualitative and quantitative exploration showcases IC_*T*_'s ability to propel system dynamics, offering crucial insights for sustainable smart grid systems. By emphasising the significance of understanding intangible measures in engineering systems, this research contributes to innovation and performance improvement in SoSE.

## 1 Introduction

This study explores the integration of intangible value logic measurements derived from the Intellectual Capital Thinking (IC_*T*_) approach to advance System of Systems Engineering (SoSE). By emphasising the significance of understanding intangible measures in engineering systems, this research contributes to innovation and performance improvement in SoSE. Qualitative and quantitative exploration showcases IC_*T*_'s ability to propel system dynamics, offering crucial insights for sustainable smart grid systems against the phenomenon of energy loss. Explaining the ups and downs of energy loss has been the work of many researchers for several years [[1], [2], [3], [4], [5], [6], [7], [8], [9], [10]]. Although understanding what is going on in Home Area Network (HAN) has long been dealt with, there is still so much uncertainty about the issue of energy loss because we know few possible approaches to understanding end-users behaviours. Meanwhile, we agree that what is happening in a complex system such as an electrical grid system could be permanently felt through incurred energy loss resulting from the electricity demand of end-users. Sharing this view, the sustaining process remains an essential task to regulate nonlinear dynamic behaviours resulting from within the home during the task of electricity demand. Management of self-sustaining societies and economies has become broadly accepted since the 1980s and has exhibited a backdrop for administration and policy decisions [[9], [10], [11], [12]]. Sustainability-based approaches to HAN were recently proposed to promote appropriate models of determining suitable strategies against the problem of energy loss. Managing self-sustaining approaches in subsystems brings about less operational complexity [9]. This scenario aligns with current thinking about electricity consumption in our daily lives and the future [10]. Embracing the principle of home sustainability, along with the goals we aim to achieve, is guided by ideas and frameworks rooted in complexity systems science. The theory of complexity supports the notion of ‘emergence’, ‘system’, ‘adaptive’, ‘dynamic’, and 'heterogeneous' elements. Other contributions to complexity theory share a belief that universal principles underlie the behaviour of systems, but they are not the same [13]. In our view, Complex systems adopt the concept of collective effect as much as its need for the power of exclusivity in practice, admitting individual systems act exclusively by setting appropriate entry standards. Therefore, we propose studying the complexity through the eyes of the three-value logics of the IC_*T*_ to support the system's sustainability, observe possible solutions, and achieve improved modelling [14]. Then, the complex notion of this problem may become clearer to control and command.

In the domain of grid systems, the focal point primarily rests on the various arrays of individual households whose interactions cause energy loss. This impulsive and hidemstic nature scale of the energy loss emerges from plenty of tiny dots where end-users passively set the odd roles, and other elements of the grid system are being influenced and followed. Many electricity end-users are good at exploiting resources but much less adept at consuming only the amount of electricity they need. Recently, electricity consumption has escalated the complexities of demand in societies. Together with an increase in the rate of nonlinear dynamic behaviour, this critique implies that a singular cause comes purely from the end-user's behaviour. Therefore, the need to facilitate the evolution of these challenges has become urgent [9]. Consumers of electricity are suffering the strong effects of the expense of coal, gas and diesel fuel; these costs are killing off the demand for those energy sources [10]. Energy costs in Australia per capita are likely to rise as the population increases, just as operational difficulties and the costs of green technologies will drop. Electricity providers will presumably develop their environmental awareness as time passes, and government incentives are likely to be introduced. A tipping point will come soon where the independent network at a household level makes for a more economical and environmental sense than a grid-powered one [11]. However, the current constraints of resources and the lack of appropriate real-time communication at a household level have brought about several performance challenges [12].

The demand side of electricity has, in recent years, suffered greatly from a range of pressures due to environmental, economic, and regulatory demands. However, optimisation of demand and supply of electricity can be achieved via development strategies that are mainly compatible with ecological and economic sustainability. While a broad range of technologies exists in electric grid systems, there is a lack of directive guidance to develop and facilitate efficient levels of using them against the non-linearity of human consumption behaviours. In the future, the demand for electricity should contribute to defining a more efficient way of optimisation and appropriate change in the design thinking of demand side management (DSM) [9]. It has become increasingly clear that the nonlinear dynamic of end-users should be controlled where possible by the capacity and time using renewable energy [9]. This involves an appropriate choice of renewable energy capacity, solar and batteries, and improved timely integration of both to enhance gains through better thoughtful specification and control strategies.

The issue is that the demand side of electricity still has no particular implicit rules around how households can and cannot behave against energy loss phenomena influencing electric smart grid systems. It is not just the grid system affected, but individual houses themselves are buckling under the weight of a surge in electricity prices caused by the energy loss phenomena. This encountered problem still happens when end-users consume electricity as it is hard to put a figure on it. Still, this energy loss would run into millions of dollars per year annually and lead to systematic national grid-scale energy loss impacts, impacted by the full spectrum of human activity as it overwhelms the grid's system to respond. The granularity of the dissimilar nature of end-user demands cannot make us eliminate this issue on a singular basis. Despite attempts since then (decades ago) to reduce the phenomena of energy loss still, evidence of more than 7.6 % transported and distributed energy loss occurs every year. This value of losses costs around $ 210 million per year in Australia [15]. It does worry, and setting out the change term to a demand-side model is then required to resolve this matter or at least push it down to less.

Along with its history, electricity is built to keep the end-users in a passive mode, still exists and has its extension even with modern grid systems. Although a growing body of literature covers many aspects of end-user behaviour in the electricity industry, it is still widely understood that the empirical validation of demand management and optimisation with electricity supply in electric systems is limited. To date, there has been minimal investigation of the ‘Spatial and Temporal Scales within DSM. What makes it harder is that the decision to a self-sustaining system relies on the capacity to test, maintain, and create an adaptive capacitive system over time. Such a procedure requires reconciling social, technical, economic and ecological imperatives [9]. To meet end-users actions in individual houses, ' Restraining actions' are needed where the origin of energy demand is causing energy loss.

The remainder of this study is mastered as follows: Section 3 argues the literature review concerning the lack of pre-existing studies of intangible assets that are influenced by human capital. In particular, this study concerning the role of intangible assets of end-users demands driven energy loss as this approach is unstructured and unrecognised yet. Section 4 sheds light on the research gap, whereas section 5 describes the set-up of the qualitative and quantitative techniques via flowchart to reappraise the mental model of demand side management by using Intellectual capital thinking values. Section 6 introduces a fresh perspective that extends beyond existing tangible assets toward intangible assets in an objective sense to address systems deficiencies. Sections 7, 8 conceptualise and present the influence of the three intangible values that cause energy loss with the formulation of value chain, value shop and value network models. Section 9 provides the causal home/demand loops to understand the causal flow and possible challenges caused by end-users demand events, along with the necessary input data and assumptions of intangible values to carry out the analysis. Sections 10, 11 provide value logic methodology and limitations. The final section, twelve, outlines the study's findings, shedding light on both its conclusions and offering insight into future recommendations.

## 2 Related works

On the matter of how energy loss occurs and what the potential intangible measures are to stop it, this IC_*T*_ study found room for improvement. Specifically, the study suggests that there is room for enhancement from the perspective of intangible system resources. The emphasis is placed on controlling actual failures that need to be detected and fixed. It has long been a concern of many researchers, and the subject of recent observations is that many efficient complex systems do not necessarily need long loops since the traces of the users are very treelike, with comparatively short loops [16]. As of now, no model has captured the concept of IC_*T*_ to address the issue of energy loss within households' individual scales (short loops) [17]. The challenge arises from the absence of a model capable of fine-tuning the demand of different consumers on the electricity grid network to achieve optimal outcomes, thereby reducing the impact of energy loss on the grid system.

**Fig. 1:**
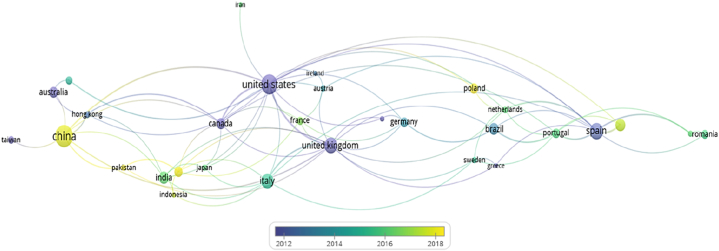
Highlighting countries concern intangible assets studies.

Numerous industrial and organisation groups in different countries are closely scrutinising the influence of intangible assets and their impact on human capital, as depicted in Fig. 1. This study, objectively speaking, introduces a fresh perspective that extends beyond existing tangible assets toward intangible assets in an objective sense to address systems deficiencies. Reviewing 43 journal papers referenced [[18], [19], [20], [21], [22], [23], [24], [25], [26], [27], [28], [29], [30], [31], [32], [33], [34], [35], [36], [37], [38], [39], [40], [41], [42], [43], [44], [45], [46], [47], [48], [49], [50], [51], [52], [53], [54], [55], [56], [57], [58], [59]] in Table 1 serves to validate the significance of “Intangible assets” and highlights its significance. Out of one million journal papers through the MDPI platform, only 118 were explicitly found addressing certain roles of intangible assets [19]. Notably, China, Malaysia, Poland, Pakistan and Russia have been at the forefront of this research topic, with China being the most. Meanwhile, Spain, UK, US, Australia and Canada were actively involved during the last decade, with the US being the most engaged.

**Table 1:** MDPI literature review of intangible assets.

**No.**	**Journals**	**Intangible Index**	**No.**	**Journals**	**Intangible Index**	**No.**	**Journals**	**Intangible Index**
1	**Sustainability 2022, Romania**	Airline companies [18]	16	**Sustainability 2022, Greece**	Intangible patenting for containerised liner shipping [33]	31	**Sustainability 2020, Europe**	Carbon Footprint Calculation and Sustainable Innovation in Intangible Assets [47]
2	**Mathematics 2022, Slovakia**	Companies' market value [19]	17	**Risk and Financial Management 2022, Czech Republic**	ICT of Knowledge- based society and knowledge-based economy [34]	32	**Water 2020, Spain**	Cost of Water upon Rates in Energy Exchanges in the Hydroelectric Industry [48]
3	**Sustainability 2022, Spain**	Discovery of hidden process data's knowledge assets [20]	18	**Sustainability 2022, Italy**	Intangible capability social, economic and environmental relationships [34]	33	**Sustainability 2019, Spain**	Intellectual Capital: Sources of Competitiveness and Organisations' Management [49]
4	**Sustainability 2022, India**	Cloud computing and knowledge management Systems [21]	19	**Sustainability 2022, Spain**	Governance Disclosure and ICT Efficiency [35]	34	**Sustainability 2019, Indonesia**	Intangible properties in the healthcare supply chain to ensure stakeholder satisfaction [50]
5	**Sustainability 2022, US and UK**	non-tradable, intangible assets [22]	20	**Axioms 2021, Mexico**	Valuable intangible asset of the human resource department [36]	35	**Sustainability 2019, China**	Intellectual Capital, Knowledge and Innovation Performance: in the Chinese Construction Industry [51]
6	**Sustainability 2022, Romania**	Intangible assets of the electricity and gas industry [23]	21	**Tourism and Hospitality** **2021, Rwanda- Uganda**	ICT of household resiliency and subjective well-being [37]	36	**Systems 2019, Peru**	An Intangible-Asset Approach to Strategic Business-IT Alignment [52]
7	**Open Innovation Tech., Market & Complexity 2022 UK and EU**	R&D investment and R&D innovation success [24]	22	**Sustainability 2021, UK**	Digital Transformation [38]	37	**Sustainability 2019, Italy**	Prioritisation of Strategic Intangible Assets in Make/Buy Decisions [53]
8	**Merits 2022, Ghana**	ICT impact and intangible value on non-financial firms [25]	23	**Social Sciences 2021, Portugal**	Hosting the Olympics represents intangible assets for host communities [39]	38	**Sustainability 2018, China**	Intangible Assets for Operating Efficiency of China Automotive Firms: 2012–2016 [54],
9	**Economies 2022, Malaysia**	Maximise Intangible assets via the speed of innovation [26]	24	**Mathematics 2021** **Commonwealth countries**	Mathematical Modelling for small and medium enterprises [40]	39	**Resources 2016, Australia**	Intangible Costs impact of the project on the visual landscape in Australia [55]
10	**Energies 2022, Poland**	ICT for environmental development [27]	25	**Risk and Financial Management 2021, Italy**	Intangible assets deriving from family business in the digital age [41]	40	**Heritage 2023, Greece**	Unrecognised part of intangible resources-built heritage assets [56]
11	**Sustainability 2022, Thailand**	Business incubators for intangible support [28]	26	**Economies 2021, Spain**	Intangible Assets and Labour Productivity Growth [42]	41	**Risk and Financial Management 2022, Poland**	Intangible nature of the digital ecosystem [57]
12	**Mathematics 2022, Slovakia**	Intangible ICT for global value chains [29]	27	**Risks 2020, Russia**	Intangible Assets of International Valuation Standards [43]	42	**Administrative Sciences 2022, Spain**	Intangibles are immaterial aspects and strategic firm resources to create sustainable value [58]
13	**Sustainability 2022, China**	ICT to improve sustainable corporate development [30]	28	**Sustainability 2020, Portugal**	Global transformation of the economy, driven by Intangible assets [44]	43	**Businesses 2022, USA, Canada**	Branding is an intangible business asset [59].
14	**Sustainability 2022, Spain**	Intangible liabilities Companies create [31]	29	**Sustainability 2020, Spain**	Intangibles into the Spanish start-ups to improve economic sustainability [45]			
15	**Smart Cities 2022, Japan**	Intangible assets for municipal value creation [32]	30	**Sustainability 2020, Poland**	Intellectual Capital of Calculating Creating Value in Agricultural Entities [46]			

Utilising the sampling size tool with a 95 % confidence level and a 10.0 confidence interval, 43 articles were gathered and reviewed, as shown in Table 1. Remarkably, the amassed articles pertain to a relatively recent timeframe, particularly the last decade, covering the period from 2016 to the present. Within these collected articles, scholars hailing from over twenty-three countries and spanning five continents conducted extensive exploitation of the intangible elements, examining different systems in various conditions and fields. Despite attempts made to define and frame the intangible elements, its nature predominantly remains unrecognised and largely unstructured. This uncertainty inherent in the intangible elements, particularly surrounding human capital systems, underscores the need for careful consideration, given its potential influence on system deficiencies and shortcomings of performance.

## 3 Research gap

This study contributes to the fields of complex project management and system of systems engineering. This study aims to bridge critical knowledge gaps and pave the way for a more comprehensive and nuanced understanding of the intricate interplay between tangible and intangible elements within complex systems. The basis of IC_*T*_-driven value creation shifts from solely tangible resources to a combination of tangible and intangible resources. On average, the benefits derived from IC_*T*_ have been estimated to be three to four times over its non-IC counterpart. Thus, it becomes vital for different organisations to understand the process involved in how the IC is created, measured, managed, and evaluated [60]. The main struggle is managing the IC_*T*_ resources efficiently for value creation. Consequently, the value added by every tangible and intangible resource must serve as an efficiency indicator, where the performance of organisations would be expected to mirror aggregate IC_*T*_ [61].

Accordingly, this study takes the initiative to search for the implications of intellectual capital thinking and analyse the influence of intangible assets with three intangible values:

• value chain,
• value shop, and
• value network.

While also taking into account the influence of tangible assets, i.e. monetary, physical, etc. Different intangible values focus on the various aspects of end-user behaviours in terms of electricity demand profiles [62]. It is expected that considering intangible assets contributes to the following:

• Reduce energy loss-driven costs.
• Define new approaches to loss-driven cost allocation.
• Define the feasibility of the intangible asset-driven energy loss.
• Select command and control approaches that result in the lowest energy loss and costs.

## 4 Materials

Essentially, this study aligns with the theoretical framework established in Refs. [9,10] to employ an IC_*T*_ approach within a System of Systems (SoS) framework. In this context, a system is a constituent of SoS comprising numerous independent elements in context as part of a larger, more complex system. Simultaneously, broader interactive systems are a group of interrelated, interdependent, interconnected systems, their parts forming cohesive entities.

The values of IC_*T*_ are explored numerically for the sake of improving SoS management and SoS measurement. Thus, our method uses an IC_*T*_ model as a second strategy class suited to the adapted case via this study that relies on competitive forces and internal and external paradigms that lead to profitability. Following this, proposing a holistic framework of intellectual capital thinking-system dynamic (IC_*T*_-S_D_) module to display the inner logic applications and capture the structural linkages among tangible resources of suppliers, retailers and end-users of electricity along with the introduced function of the three intangible values: (1) value chain logic (V_CL_); (2) value shop logic (V_SL_) and; (3) value network logic (V_NL_). This implies that the internal structure will be verified, designed, tested and developed according to a particular grid objective. This work of using value logic thinking via IC_*T*_ helps to provide a much bigger mirror than we have ever had on the influence of energy loss. It lets us get higher resolution looks in a much more feasible way and a shorter observation time.

**Fig. 2:**
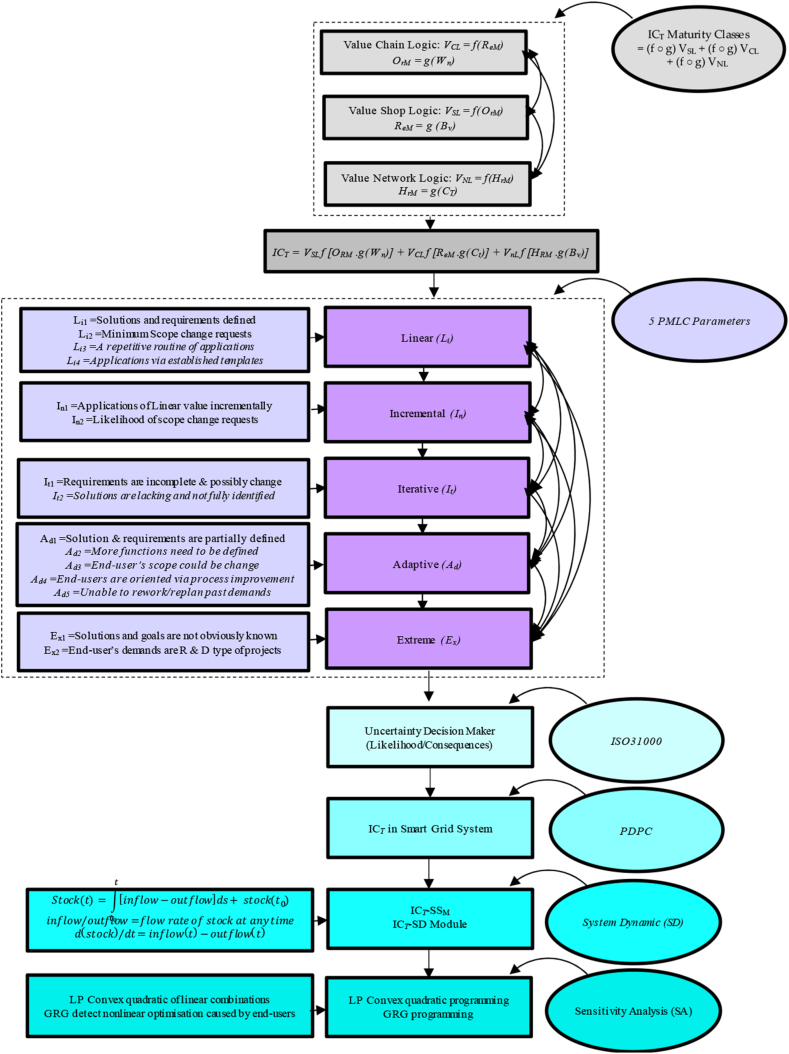
Flowchart of IC_*T*_ approach.

We use the light of the study flowchart in Fig. 2 to cluster dynamic data of DSM in electric smart grid systems to generate models for state estimation that support the goal of systems' performance. Mathematically, we have simulated the results in this study by using six qualitative and quantitative methods: (1) IC_*T*_ logic values; (2) Process Decision Program Charts technique (PDPC); (3) Five project management life cycle (PMLC) parameters, (4) ISO31000, (5) system dynamic (SD); and (6) sensitivity analysis (SA). The PDPC technique has been used to visualise the steps required to achieve the IC_T_ objective. Following this, applications of SD are adapted from the source [17] to draw a causal flow diagram and determine how a household is relevant to the practice of self-sustaining governance. To complete this work, necessary adjustments were implemented to re-evaluate the structure and create concise loops using the loops of six DSM-based homes from the Causal flow diagram. This process aims to propose a sustainable management system incorporating feedback loops aligned with IC_*T*_'s objectives. These proposed mixes of qualitative and quantitative testing methods are based on the variance decomposition of IC_*T*_-S_D_ module responses. The consequence of this integrated analysis approach is to excel managerially but not inevitably cultivate the desired strategic result. In this context, a total of 10 million data items were gathered and associated with identified residential end-users within locally sourced 250 residential houses, utilising an annual data calendar structured around 30-min intervals.

## 5 Intangible assets

The intangible studies style demonstrates a consistent correlation between intangible assets and elevated performance levels [63]. However, intangible assets receive minimal attention from scholars, primarily because of its heterogeneous nature and minor amount of influential resources compared with other tangible resources. Nevertheless, the significance of intangible assets is increasingly recognised in future investments, and research and development efforts offer valuable insights for decision-makers in related fields [64]. With regard to the knowledge-based contemporary, intangible assets recognised as non-traditional assets qualified to derive interrelated values within a spectrum of integrated activities. Multiple decision models can be created to incorporate the benefits of intangible resources, adding greater flexibility. Intangible assets intellectually spot dysfunctional behaviour in a group of stakeholders, particularly subjective behaviour that is hard to assess within a tangible framework. The technical challenge of intangible assets is a behaviour-focused, highlight an opportunity to tackle organisational issues. Implementing recognised metrics introduces an objective approach, enabling solutions and foster better alignments among stakeholders [65]. Intangible assets are akin to goodwill in enhancing future earnings performance, and they have neither financial incorporation nor physical substance. The essence of intangible assets lies in its core objective of contributing to forecasting to improve the overall system profitability [66].

The notion of investments in intangible assets is motivated by better management and better resource assessment of a system's sustainability and value generation. Perhaps intangible assets are in place with tangible assets of an existing working system. Concerning the applicability, the issue associated with valuing intangible assets provides adequate value estimates to develop more formal and comprehensive tools. However, constraints are anticipated, which may deter the generation of more information on intangible resources, which is likely to be unwelcome due to the difficulty of the information being understood or perceived for decision-making. The relative challenge of clearly defining intangible assets ought to highlight weaknesses where the former loss occurs and most likely requires new investments via a credible intangible asset. Put differently, the matter of Intangible assets is outlined in conjunction with a justification for a suitable methodology of valuation. Intangible assets are not physical entities that possess a particular recognisable description and identification. The value of intangible assets is determined through deficiency gaps after losses are defined and incurred within a system. The intangible assets are identified by first understanding the system value drivers that shape and constitute them. Once the value drivers are acknowledged, the mingling and mixing of these drivers uncover particular intangible assets that align with a system requirement [67]. More precise information about intangible assets aids in implementing suitable valuation methods [68].

Coupled with the above argument, intangible knowledge development, management, and measurements are difficult to achieve due to the lack of essential monitoring-related systems, feedback loops and then formalised intangible systems. Consequent to the probability of failure of future activities, intangible values mimic that to ingress appropriate changes tend to render more successful applications. The current low-reporting operational environment proposes to enhance information concerning the usage and holding of intangible resources, increasing the potential of intangible values of such reporting to stakeholders [69]. Thus, the benefit of extensive reporting among stakeholders involves significant investment in intangible assets with fairly uncontroversial, efficient management. In summary, intangible assets could be introduced as an audit tool to drill down, capture and prioritise values based on know-how, Show-how, Explore-how, and exploit-how, thereby fostering the creation of new values.

## 6 Results maturity classes of IC_*T*_

Considering an SoS, the complete resources classification system embraces two relevant classes of strategies, one class grounded to the industry's portfolio (i.e., smart grid system) where the argues variance in profitability as a matter of holds or lacks valuable resources. The second strategy class relies on the internal and external paradigms of competitive forces that lead to profitability, which highly depends on change management and strategic decisions that alter the firm's ability. This work examines DSM, applying generalised states at different inference levels and utilising an IC_*T*_ model to produce practical outcomes derived from grounded concepts of causal relationships based on existing theory. The bringing forward of IC_*T*_ properties expected benefits from using several values conceptualisation. We floated the possibility of IC_*T,*_ perhaps boosting a large-scale advance procedure in the electric smart grid as what we know in this study area is still under development.

Previous studies described energy loss based on holistic, systemic viewpoints. Some other studies with a narrower focus provide underpinning evidence for limiting the grid's tangible resources. Because of energy loss due to the current trends in the global energy demand, IC_*T*_ value logic scenarios are expected for the future grid system to introduce a great deal of attention to improve grid performance. One of the main assumptions formed in this study is that besides the common gap of deficiency made by tangible resources (physical and monetary), there is also a possibility of increasing or decreasing this gap by other intangible resources centred synchrony.

**Fig. 3:**
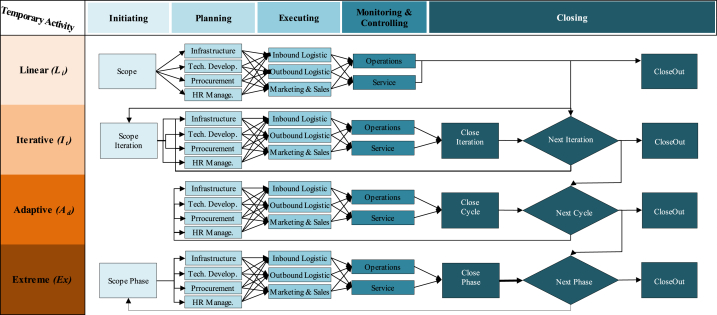
Value chain logic [69,71].

**Fig. 4:**
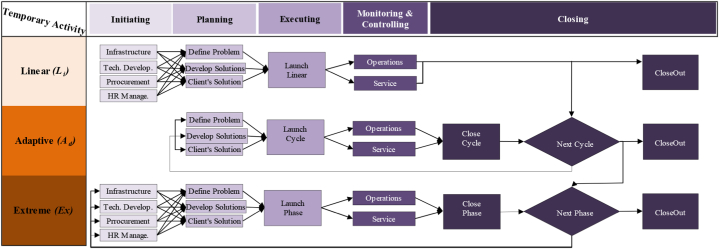
Value shop logic [70,72].

**Fig. 5:**
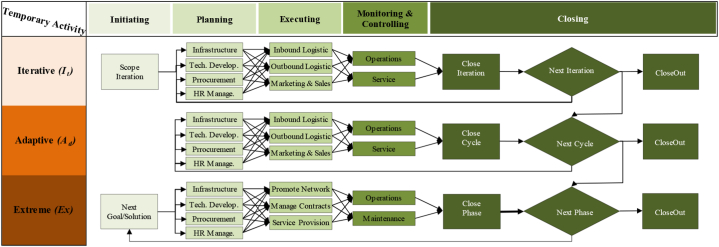
Value network logic [70,72].

In this study, the IC_*T*_ idea was adapted from Ref. [9]; divided IC_*T*_ into three maturity classes: Relational (R_eM_), Organisational (O_rM_), and Human (H_rM_). R_eM_ class implies all the stakeholders' activities, i.e., suppliers, regulators, retailers, end-users … etc. O_rM_ class represent all the firm's assets, excluding human resources, and we can introduce it via anything not being a part of the balance sheet, i.e., procedures, policies, brands ….etc. The third class entitles H_rM_ and is defined as a non-replaced agent, i.e., attitude, competency, skills, behaviour, beliefs … etc. Going straight to the point, IC_*T*_ makes sense in the functional/bottom line at any company and gives sense to the value of the employee's skills, experience, and knowledge to support the competitive advantage features against other competing firms in a market segment. The trade-off of IC_*T*_ implies controlling nonphysical/nonmonetary resources in an organisation's value creation. IC_*T*_ is not displayed quite often or even accounted for throughout a system execution or delivering an immediate sense of a system performance similar to the tangible resources such as technology, physical and monetary resources attributions. In addition to this, the five features of Wysocki PMLC in Fig. 3, Fig. 4, Fig. 5 are used in favour of IC_*T*_ applications for each value logic to Compare their rational architecture to the norms of Linear (Li), Incremental (In), Iterative (It), Adaptive (Ad) and Extreme (Ex) models [70]. Thus, the attempt made in this study is to define IC_*T*_ as a value generation machine to parametrise the way resources are managed. This study focuses on the technique of IC_*T*_ and concerns the following three spectrums: (1) value chain logic (V_CL_), (2) value shop logic (V_SL_), and (3) value network logic (V_NL_).

### 6.1 Value chain logic (V_CL_)

The supply chains of electricity in the developing world are moving to sustainable power sources away from the stations of supply generators and reducing the overwhelming sense of global responsibility. Electricity cost transactions are continuously monitored by prices. Unequal to other industries, electricity net demand is not influenced by inventories held at points along the way of the supply chain (see Fig. 3). The prospect of V_CL_ is when both trends of suppliers and consumers alike converge to avoid the integration of spillover benefits and embrace the integration of utility maximisation. The perception of sustainability incorporates science as we have come to know it and pushes the boundaries of achieved technical knowledge into the realms of social policy and philosophy [71]. Certainly, suppliers, retailers and end-users are all in agreement on environmental concerns. However, suppliers and retailers are more concerned about decreasing generation power losses to secure more benefits. Meanwhile, end-users are concerned about decreasing the payable bills of electricity. By the same token, the promised benefits of activating self-sustainability approaches at household levels are mainly to enable, settle and combine both parties to maximise their benefits equally.

It is essential to mention that both suppliers and retailers have similar trends; when retailers generate a higher expected profit, suppliers' profit declines, and vice versa. Subsequent to what has been reported, expected that the total chain profit of the suppliers and retailers remains almost unchanged. The trading of energy among supply-demand stakeholders operates by an independent system operator where each stakeholder leverages its goal of minimising the cost for maintaining energy balance. In most cases, the electricity cost is considered after end-users have consumed it and received a bill for the consumption period. Most end-users pay a bill that mirrors the average cost over a period, often over a quarter or a year. With this, there is a conflict against the demand management options for short-term use related to cost-reflective prices.

The current problem, the empowerment development nexus at electric smart grid systems, discussed the interrelationships of the scale agents and found too weak to be self-sustaining [72,73]. Sustaining a system is more than illustrating the coordination of systems' components; it is the exchange between energy and matter (human), where complexity awards stability [74]. These patterns attribute to the spatiotemporal memory of individual agents, where increased complexity factors should come at the cost of increasing the stability of the value chain logic of a system [75].

The intangible logic chain complex issue is industry-dependent and arises because the suppliers and buyers (retailers and end-users) make their self-interested consumption, production and pricing decisions [76]. This fact reflects that both downstream (supply-demand) and upstream (demand-supply) of the value chain logic, in a binary monopoly, simultaneously apply their market power against each other. This continuously experienced attitude of the V_CL_ will negatively influence the equilibrium of the supply-demand chain of electricity and lead to departing from the expected standard course. The vision of adopting V_CL_ open to all stakeholders provides an opportunity to deal with one agreeable objective to improve the chain's efficiency.

Developing the household network for sustainability growth needs to move further than the type of claims typically set out to influence isolated factors [77]. Our objective in drawing on value chain logic (V_CL_) is to develop a practical home perspective that relies both on continuity and change. A suitable proposed process for the home should indicate which conditions need to be in place to deliver the emergence of sustainability and make these homes separately able to change [11]. It means a network's sustainability depends on the extent to which household utilities are nurtured and maintained over time, which eventually increases the sustainability of homes. This is influenced by interdependent relationships of consuming behaviour complexity and how the household is being optimised at the local level [77].

Realistically, there is a connection between the electricity supply chain from the generation to the demand side, but there is a lack of connectivity from the demand to the supply side. Supply chain management of electricity is a timely integration of power generation, transmission, and distribution in a number of substantial deterministic processes to cover uncertain demands [74]. Therefore, the role of V_CL_, through the perception of OrM, is to enforce the supply chain of electricity, which considers the instantaneous activities of consumers to remain optimal against energy loss phenomena and to respond timely and appropriately to the changing behaviours of end-users.

### 6.2 Value shop logic (V_SL_)

The role of V_SL_ contributes substantially to electricity stability and the reduction of electricity loss. Electricity retailers are jointly working together with suppliers, end-users and other utilities to derive smart grid systems with the following advantages: 1) High, consistent service; 2) Highly reliable service; 3) Practical, proper management to derive efficient energy; 4) Self-healing facilities; 5) Automated systems and accurate decision; 6) Support systems; 7) Smart metering integration, and 8) A very high penetration of renewable energy [78]. This objective of V_SL,_ concurrent with the vision of the 21st century, increases the grid's stability by managing losses caused by the instability of the retail electricity market at the national grid system level.

Although several studies have discussed the trade-off issues in the retail electricity market where V_SL_ belongs, there is a gap in understanding the role of V_SL_ in improving this nonlinear dynamic mechanism, particularly in electrical smart grid systems. The basis of V_SL_ has an inherent management focus on the system's effectiveness rather than on the system's efficiency and aims to solve problems and produce unique solutions, which is defined as an ability to reconfigure the system resources continuously (see Fig. 4). Thus, V_SL_ has effectively centred on electricity retailers to reduce the internal coordination cost of a system that emerged qualitatively from behavioural reasons of electricity demand (end-users).

There is a strong correlation between the energy supply and demand. The grid system wellness is the responsibility of caring value shop logic, which can strategically position the reluctance of systems' resources against energy loss as helping stakeholders achieve financial wellness and go beyond just offering products to them. The desired approach to home sustainability requires quick adaption to individual changing circumstances. Autonomous sustainable systems rely on the intangible power of V_SL_ to provide a collaborative and iterative learning approach to generate continual innovation and, in addition, respond to rapid environmental changes [10]. When considered in the context of complex systems, the approach of value shop logic is itself a complex activity, and the trading power of complexity lies in the shift from linear to exponential orders of magnitude [79]. Therefore, the intellectual capital thinking of V_SL_ is revived by facilitating great research skills to think, contribute, and coordinate with new effective solutions. The tangible monetary and physical resources of electricity suppliers and end-users are the basis for V_SL_. However, the fact is that the intangible resources of VSL localise in the virtual zone of electricity retailers, where the trade-off is leveraging the price-cost.

In the context of the current risk of energy loss and with the ongoing behavioural constraints of peak and off-peak electricity demand, the role of V_SL_ strongly considers the benefits of earlier action with the same in-use resources rather than looking for alternative solutions that need further resources not available in the system. To conclude, the product of V_SL_ is a pivotal source that helps stakeholders thrive equally in a competitive market, minimising the interest conflict between electricity stakeholders. Likewise, this goal aligns with the national target of controlling and mitigating air emissions waste to reduce the influence of climate change.

Traditional resources, while necessary, are not by themselves sufficient for understanding and dealing with the case of intellectual capital thinking. The value shop logic, called electricity retail, is complex, diverse, and dynamic [14]. There is a concern about the scientific uncertainty surrounding the management of electricity consumption activities. The decision-making process remains uncertain about how far the retail of electricity will run. In the longer term, the perspective of traditional resources creates more time for the context of the system to make a change [10]. The observer of the value shop logic is entirely determined by its approach, which would be understood in the most basic way [80]. Exploring the rationale of underlying systems thinking and system applications requires more attention to self-sustaining principles [10]. Therefore, a value shop logic characterises its observation of V_SL_ as the process of interaction between the two predominant coherent structures (renewable and traditional energy) with the possible additional functionality of a domestic scale to cover its space demand [[81], [82], [83], [84]].

In a practical manner, electricity retailers face the volatility risk of spot prices rising to levels at which they have no option but to pass increased prices on to customers. Oppositely, generators face the volatility risk when low spot prices occur, leading to reduced average earnings. Relational maturity (R_eM_) can simultaneously measure the agility of electricity power (asset) being available to complete a demanding task. V_SL_ focuses on the supply, transmission, retailing and electricity demand by providing specific attention to individuals and their problem-solving abilities. Therefore, the role of V_SL_ is entitled, through the perception of ReM, to reconfigure the portfolio resources and discover new issues continuously.

### 6.3 Value network logic (V_NL_)

Two cases decrease network sustainability, and those are disasters that are not common and emergencies of end-user demands, which occur every second in electrical smart grid systems [85]. The interrelationship of households increases the interaction, and the number of actors multiplies, increasing the chance of an effective network. The most critical factor for sustainability is how to provide the right concept for utilising structural factors.

While we are looking at what we can do to support the system's performance, the aggregation outcomes of a single-house network model can be caused by a series of loops and webs that make more sense to the value of intangible resources implied in the meaning of understanding a network of interacting core competencies (see Fig. 5). Understanding a network of individual households increased by extending what is already known about electricity demand through an iterative process that can be achieved from a retrospective picture of technical demand data [10]. However, when examining the V_LN_ of the two different households/end-users and looking at the various types of consumption they are practising on a daily basis, the difference between them will be visible [14]. Increasing this unique knowledge for each end-user is a chance creation for radical changes that can be attributed to adding Value Logic Network (V_NL_). From a macro network level, the supply of smart grid systems would act solely as a conduit to arrange the demand flow to and from micro-networks of end-users.

As always, at a household level, it will continuously exist more amorphously between new emerging power events and those that already exist [76]. Meanwhile, the prevalent action will be sustained according to the system optimisation needed. The term ‘path dependence’, chosen for ‘sustaining purpose’, is allied to the concept of ‘illuminates lock-in’ to sustain the point where other more sufficient phenomena are competing, resulting in a redirection of that path. From a Physics perspective, a household system will be sustained in specific ways by excluding the weaker agents that are further weakening themselves in an endless and vicious cycle [77]. Importantly, value network logic (V_NL_) takes fully exposing individual observers for each household to realise the complex image of societal self-sustaining under the constant control of the regulatory intervention. The observation of the self-sustaining process is a merited expression if only to lay out the groundwork adequately toward the contributions, such as the one feed, to add consciousness through the monitoring and controlling systems in various households.

V_NL_, the network activity can be found in societal tiers in electrical smart grid systems when more people engage in a reciprocal exchange (e.g. feed-in tariff). Therefore, sets of feedback loops for individual household systems are crucial and relevant to the perception of value network logic (V_NL_) where nonlinear causal models is adequate to describe what goes on in a small-scale system. Therefore, the first aspect of building an objective functional model of V_NL_ for self-sustaining involves framing the smart grid system at the household area level and mapping what is possibly involved and the essential relationships used to define the system. It describes the phase of the dynamics of the consumption scenarios that should be developed. As a result, the compilation structure gained from the V_NL_ rehearsals shows how such a situation could unfold in the future [10].

In electrical smart grid systems, the demand of end-users is proposed as autonomous agents, which must meet with approaches of multi-issue negotiations. However, empirical evidence assumes that autonomous agents are often what individual (network) end-users in grid systems are, as individuals often fail to formulate possible joint gains and finish with inefficient integrated systems. In this sense, end-user/home demand networks display one of the most essential phenomena to be traced and controlled. Therefore, developing V_NL_ strategies is required in individual households to support reaching an efficient desired goal [84].

## 7 Summary of value logic review

Taking into account that the greater population in the grid system exists in HAN, it shows a short-term demand for purposes that can be considered relatively and immediate; it makes the concept of sustainability harder to trade, and the greater the incentives to illustrate reciprocal exchange relationships [86,87]. Also, the interface amongst individual levels of households and their systems to initiate second-order change occurs at that micro-level within a macro smart grid context. The relevance of system complexity at micro levels crystallises around the dynamics involved in the interfaces between multiple individual house levels and the electrical smart grid context [36]. The volume and quality of IC_*T*_ are asymmetrical deliverable resources, uncertain and not traded resources. The point is that IC_*T,*_ on a continuous basis, leads to inefficient outcomes and affects the total system's performance. In addition to this, on the IC_*T*_ role, evidence was led that the smart grid system acknowledges these three forms of O_rM_, R_eM_ and H_rM_, implied through three values phenomena named V_CL_, V_NL_ and V_SL_; these forms are defined as ‘ownership’ (W_N_), ‘control’ (C_T_) and ‘behaviour’ (B_V_) respectively. Based on these facts, governing the electricity demand of end-users revolves around massive and sudden or incremental and gradual intangible behaviours [86]. Therefore, capturing these concepts in a framework means focusing on critical interfaces elaborated through specific models and processes of tangible and intangible values [88]; please see Table 2.

(1) IC_T_ = (f.g)) V_SL_ + (f.g) V_CL_ + (f.g) V_NL_

(2) *IC*_*T*_ = *V*_*SL*_*f [**O*_*rM*_*g(W*_*n*_*)]* + *V*_*CL*_*f [R*_*eM*_*g(C*_*t*_*)]* + *V*_*nL*_*f [**H*_*rM*_*g(B*_*v*_*)]*

*Given values V*_*SL*_*, V*_*CL*_*,* &where's

**Table 2:** PMLC parameters versus IC_*T*_ values logic.

Models	**Parameters**		**V**_**CL**_	**V**_**SL**_	**V**_**N**_**L**
PMLC	Linear	L_i1_ =Solutions and requirements defined		✓	
		L_i2_ = Minimum Scope change requests			
		L_i3_ = A repetitive routine of applications	✓		
		L_i4_ = Applications via established templates	✓	✓	
	Incremental	I_n1_ = Applications of Linear value incrementally			
		I_n2_ =Likelihood of scope change requests			
	Iterative	I_t1_ = Requirements are incomplete and possibly change	✓		✓
		I_t2_ = Solutions are lacking and not fully identified	✓		✓
	Adaptive	A_d1_ = Solution and requirements are partially defined			✓
		A_d2_ = More functions need to be defined			✓
		A_d3_ = End-users scope could be change			✓
		A_d4_ = End-users are oriented via process improvement	✓	✓	✓
		A_d5_ = Unable to rework/replan past demands	✓	✓	✓
	Extreme	E_x1_ = Solutions and goals are not obviously known			✓
		E_x2_ = End-user's demands are R & D type of projects	✓	✓	✓
IC_T_	Relational Maturity	R_eM_ =Relational Maturity of stakeholders	✓		
	Organisational Maturity	O_rM_ = Organisational Maturity of assets		✓	
	Human Maturity	H_rM_ = Human Maturity agent			✓
	System's Behaviour	B_V_ = Maturity of Behaviour: Suppliers, regulators … etc.	✓		
	System's Ownership	W_N_ = Maturity of Ownership: Firms and assets		✓	
	System's Control	C_T_ = Maturity of Control: skills, competency … etc.			✓

V_SL_ = f(O_rM_)

V_CL_ = f(R_eM_)

V_NL_ = f(H_rM_)

O_rM_ = g(W_n_)

R_eM_ = g (B_v_)

H_rM_ = g(C_T_)

We attempted to combine information from an intangible managerial perspective in a way that is bound to the strengths of tangible system assets to parametrise how these resources manage the ‘Sustainable Human Future’. A limitation of this study is that it focuses on the DSM, on the segment of residential households in the electrical smart grid systems. To clarify where this study is heading, we emphasise the IC_*T*_ functional role in praising nonmonetary values, where the expected benefit of the IC_*T*_ approach pays particular attention to unforeseen intangible resources to improve system performance. Adding further clarity, the risk matrix driven by international standard ISO31000 is used based on the effect of corporate governance and decisions making on objectives achievement caused by uncertainty [89]. We use these investigative matrices to draw a range of consequences and likelihood, and the probability of something should happen concerning the collective influence of the three value logics V_CL_, V_NL_ and V_SL_ on IC_*T*_. The uncertainty assessment of these matrices should describe a possible trajectory that occurs in the nature and the decision level of the logic values objectives that could affect the achievement of the IC_*T*_.

**Fig. 6:**
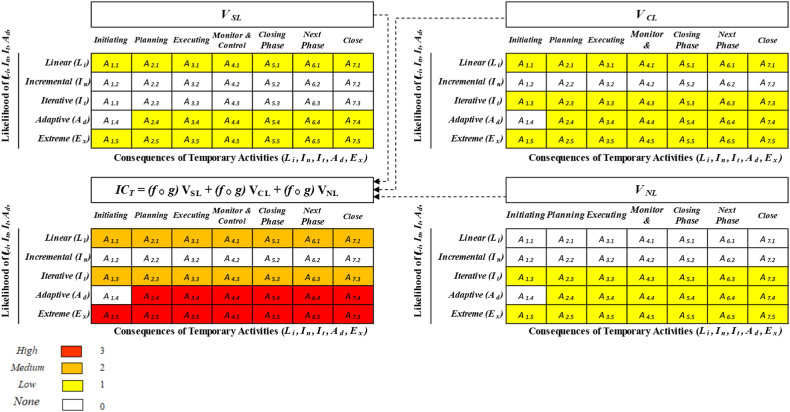
Likelihood/consequence matrices of IC_*T*_, V_SL_, V_CL_ and V_NL_

**Fig. 7:**
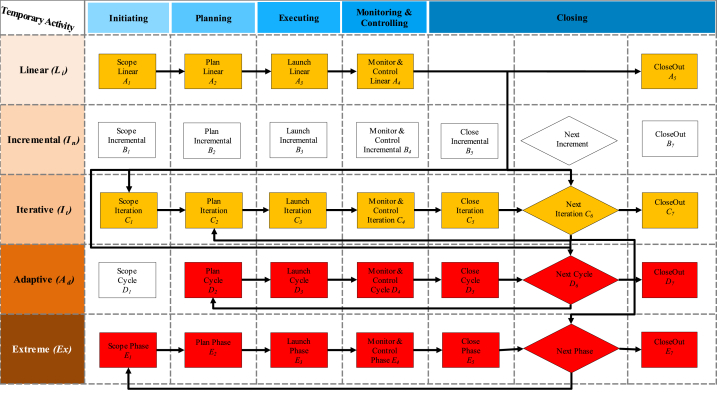
PMLC models (*L*_*i*_*, I*_*n*_*, I*_*t*_*, A*_*d*_*, E*_*x*_) Vs. Ic_*t*_ logic values (*V*_*cl*_*, V*_*sl*_*, V*_*nl*_).

From these standpoints, five consequences of typical lifecycles of five PMLC types: (1) Linear (L_*i*_); (2) Incremental (I_*n*_); (3) Iterative (I_*t*_); (4) Adaptive (A_*d*_) and (5) Extreme (E_*x*_) were used for the assessment. The relevant events consequences for each of these PMLC types are denoted from *A*_*1*_ to *A*_*7*_ (see Fig. 6), and the strength of their bias adequacy of likelihood is characterised from low to high in four colours: white, yellow, orange and red, respectively. The influence of these value logics-driven IC_*T*_ matrix achieved is expressed based on the magnitude of the combination of consequences and likelihood to generate priority and rating of possible PMLC trajectories shown in Fig. 6 above and Fig. 7 below.

Along with the combination of consequences and their likelihood provides an easily understood display of different decision-based uncertainty levels. The Process Decision Program Charts (PDPC) technique has also been used to visualise where the steps of IC_*T*_ objectives apparently build rational events. The purpose of using PDPC, display IC_*T*_ in a flexible manner that accommodates chronological dependencies and presents a broader view of the intangible values in question. The structured layers of five levels of PDPC have a systematic means of anticipating intermediate steps to uncover the link between potential areas that could cause errors to derail reaching the desired goal. The emphasis of using PDPC outlines and relatively compiles the consequential impact of three intangible value logics (V_SL_, V_CL_ and V_NL_) on activity plans. Keep in mind that the previous discussion anticipated the meaning arising from each IC_*T*_ logic value (V_SL_, V_CL_ and V_NL_) and mapped out each with appropriate information and flow charts associated with all activities and tasks that must be considered.

## 8 IC_*T*_

**Fig. 8:**
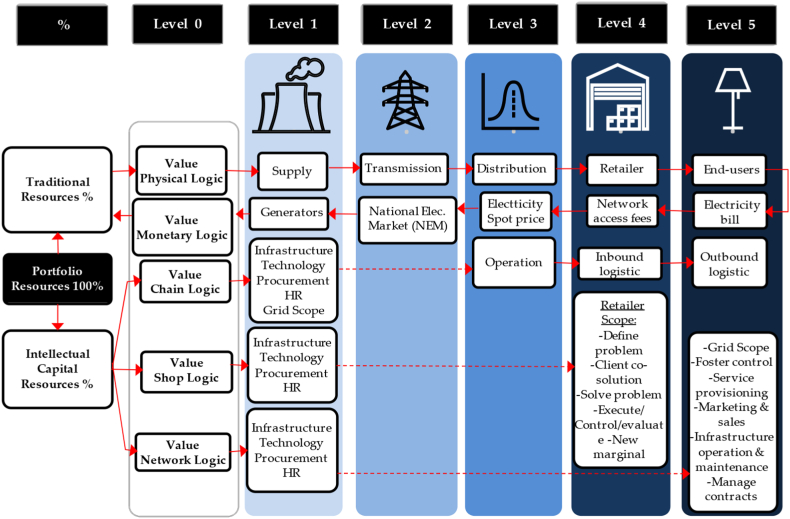
PDPC of IC_*T*_

**Fig. 9:**
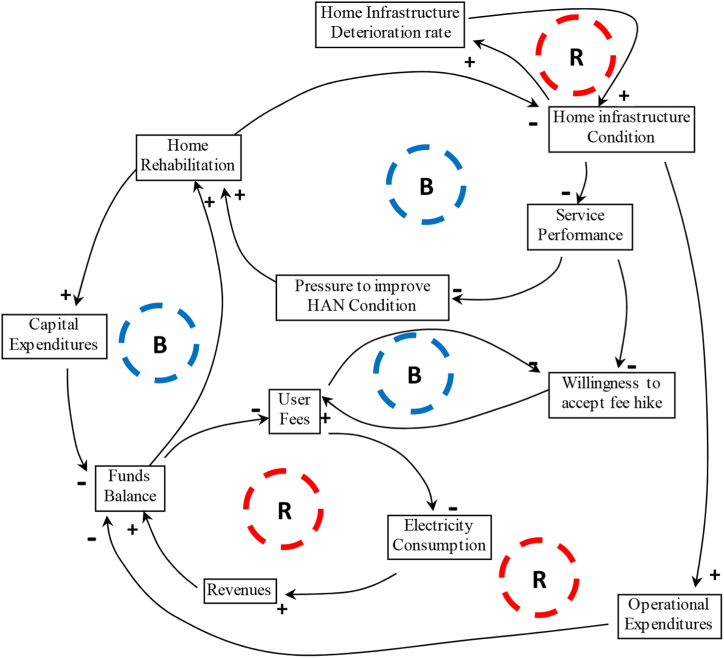
Causal flow diagram.

As notified earlier, each section of this study has shown a reframed debate about the V_SL_, V_CL_, and V_NL_ in how IC_*T*_ approaches the sustainable condition of individual home demand, which's subject to biases in design and execution. This intention is also re-maintained in this section regarding systems' sustainability via Fig. 8, Fig. 9 to understand the causal flow and possible challenges caused by end-users demand events as a value chain analysis to aid understanding of the internal context.

**Fig. 10:**
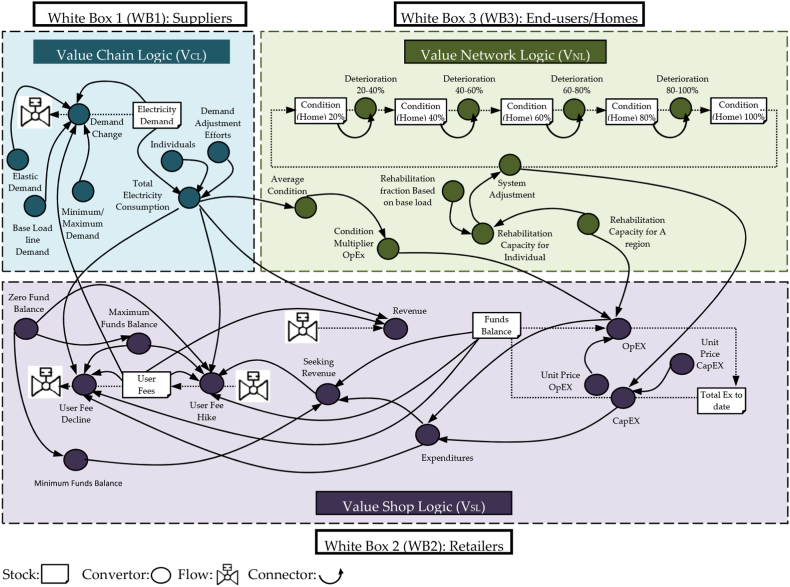
IC_*T*_-S_D_ module.

The idea of the self-sustaining model (SSM) approaches driven solutions, basically taken from complexity philosophy to propose an autonomous framework, reveals a set of technical solutions that may be implemented [13]. The causal loops and system dynamic model (SD) were discussed below in Fig. 9, Fig. 10 to propose electrical household sustainable management systems involving multiple interacting feedback loops that suit the goal of IC_*T*_. We borrowed the idea of the sustainability model from this source [17]. We also re-exploited the model to establish short-term loops (a household system) instead of long-term loops (a grid system). Then we made related changes to draw out a household relevance to the practice of self-sustaining governing. In the wake of this causal loops model, the feedback loops built based on object-oriented modelling consisted of a series of integrated scenarios jointly developed on what decisions need timely manner to be taken to build resilience suit to the sustainability approach of household area network (HAN) and linked to the network value logic of intellectual property capital thinking.

The observations were made using six DSM-based home loops. The reinforcing loop (R1) of household infrastructure deterioration shows the rate of household sustainability based on the capacity of renewable energy (solar, wind & battery), determines the condition of the homes individually. The rate of deterioration describes a particular need for renewable energy resources for a household. The timely feedback loop then reports the deterioration cases and collectively points out the possible action needed. The balancing loop (B1) is a function of R1act when energy loss occurs to provide home rehabilitation. B1 learning from R1 and home historical deterioration behaviour, which subsequently derives systems' improvement.

The reinforcing loop (R2) proposes that individual households are financially self-sustaining when the revenues exceed or equal their expenses. The electrical utility increases user fees when fund balances (revenues minus expenditure) fall to a specific threshold value. The reduction in using electricity at peak times is characterised by time delays that shift the consumption to other occasions controlled by the loop of R1. The self-reinforcing feedback loop cannot operate indefinitely since any constraints around the loop could trigger to stop growth. Changing demand behaviour of individual houses sometimes occurs where the purpose of the balancing feedback loop (B2) is to make sure that the willingness of the sustainable home system can meet the fees hike and provide more funds throughout the capacity of the sustainable home system. The role of feedback loop B2 binds the finance with the sustainable capacity of individual homes related to the objective of load profile at individual households.

The loop of capital expenditures (B3) has a specific role in investing the individual home revenue for the rehabilitation process (B1), which is influenced by the deterioration rate of loop R1. The final reinforcing loop (R3) encompasses four parameters together: Home Condition (R1), Funds Balance (R3); Operational Expenditures (R3); and home Rehabilitation. The deterioration of a household not only depends on the scale of electricity consumption and the home readability-based renewable energy, but this scale also rises and falls according to consumers' behaviours. Both scenarios incur additional costs for the grid utility unless consumer behaviours do not return operational benefits when rehabilitating the household electricity infrastructure. The typical nature of the loss occurs due to end users' nonlinear dynamic behaviour, increasing operational expenditures and reducing funds' liability for the future. When a household is less rehabilitated, the state will further deteriorate and conduct the cycle of deterioration, which increasingly accelerates.

Attempts were made to add IC_*T*_ functions in the causal loop model to align the local home system based on course-correct messaging around home sustainability objectives. A briefing drew on the micro-scale causal diagram in Fig. 9, incorporating values adapted from IC_*T*_. Quantitative data will be used for assessment in the upcoming sections. Depending on the consequences and likelihood of V_NL_, V_CL_ and V_SL_ in the causal loops might place sustained outcomes when it controls various home's activity patterns individually for the fully rehabilitated goal. Such variability of end-users’ behaviour raises the role of the value logic of IC_*T*_ to confirm the significance of home sustainability influenced by the spatiotemporal memory of individual agents (end-users) [16].

## 9 Methodology

Building upon the findings of the sources [9,10], and [71] to support the simulation modelling of the three black boxes to get a higher resolution mimic in a much more feasible way and a shorter observation time. Consequently, the picture of new observatories obtained from the analysis of electricity demand will become much more evident. The proposed practical operator method of the IC_*T*_ approach in this study is a framework that implies value chain logic, value network logic and value shop logic, and that helps to reveal the relationships of the various constructs under investigation. It belongs significantly to the theories developed from the literature, which need to be formed into a ‘theoretical framework ' as a ‘module to proceed with the subsequent practical applications. Thus, the attempts shown across the blocks in the IC_*T*_-SD module, as illustrated in Fig. 10 offers a structured approach to provide tailored responses to varying levels of demand for individual end-user systems. This complex demonstration module of a hypothetical self-sustaining home utility highlights the role of IC_*T*_ values in finding errors and potential countermeasures. To describe this phase of the module study that we are currently under, proposing the inner logic applications and referred to as a function of three black boxes A, B and C in the IC_*T*_-SD module, implies that the internal structure will be verified, designed, tested and developed according to a particular grid objective.

The correlation role of IC_*T*_ in this study to re-prioritising and re-routing a home's stock occurs purposely in individual systems of end-users to serve the optimum grid goal. To simplify this, assuming the proposed modelling will cover five conditions' groups (CG) of a household stock, those are CG-20, CG-40, CG-60, CG-80, and CG-100. Each condition group is assigned an average condition grade using an arbitrary scale that varies from 0 % to 100 % to define the capacity of each household needed to install renewable energy (stock) and to serve the approaching goal of the baseload line (a desirable demand) [17]. The proposed solution of a baseload line is a unique goal (average targeting demand) that mainly considers the end-users dissimilarity as one of the main objects of attention.

Our points of concern are discussed in various sections; theoretically, IC_*T*_ has much scurrying and flurrying displayed in many studies on the way, not practically accounted as a part of a system command, execution and control or delivering an immediate sense of a system performance [14].

Back to Fig. 10, the IC_*T*_-SD module aims to simplify the variance-covariance matrix needed to compute the optimisation of intangible resources of value logic, a complement to physical and monetary resources (Tangible resources). Therefore, the specific goal of IC_*T*_ is a demand-based objective of the grid system, retailers and end-user satisfaction. Where IC_*T*_ contributes to this module by breaking it down into three values logics: V_CL_, V_SL_ and V_NL_. The first term, Stock (S_t_), in the IC_T_-SD module, deals with two combinations of physical elements, anticipates the electricity capacity available of storage batteries (B_TC_), the capacity of used solar power (S_TC_), and the availability of other traditional sources of power generators (G_P_) at a given point of time. The term ‘stock’ also makes sense on the instant 'traces' left of end-users' demand [17]. These S_t_ elements lurk while delivering or consuming electricity. The term ‘flows’ will remain a coherent process of ‘in’ and ‘out’ measures in place based on available stock to show the activities calling for the stock through transport quantities either instantly or over time. Besides the aforementioned information, the term 'converters' (C_ON_) is also used to define the appropriate renewable energy limit at individual homes based on the unique and different ‘deterioration’ of individual demand cases (See Fig. 10). The daily consumption of a household concurrent with a need/no need rate at which renewable energy resources move from one condition grade into another. This is coupled with timely revenues and expenditures of a grid utility [17]. The rational formula between flows and stocks can be formulated and introduced as follows [89]:

(3) $$Stock\left(t\right)=\int_{t_{0}}^{t}\left[inflow\left(s\right)-outflow\left(s\right)\right]ds+stock(t_{0})$$

(4) inflow(s)/Outflow(s)=flowrateofstockatanytime

(5) d(stock)/dt=inflow(t)−outflow(t)

S=Stoc

t_0_ = Initial time

t = Current time

Stock t_0_ = Initial value of the stock

A comprehensive theoretical causal flow diagram in Fig. 9 and the dynamic system model in Fig. 10 were exhibited to tackle energy loss problems in electrical grid systems. The circle of thinking uses the lens of how to immediately enable household environments to understand and take action on the nonlinear dynamic behaviour of end-users while consuming electricity [77]. The attempt shows how using intellectual capital thinking will produce a set of heuristics that can be aggregated and applied to support the dynamic of house area networks [13,89,90]. This study attempts to define what could be a ‘home sustainable model’ in terms of the flow processes at a single-house level. The proposed conceptual model in this study is an abstraction of the way decided to comprehend a particular part or function of reality [91].

### 9.1 Black box A (BBA)

BBA is part of a broader class of the IC_*T*_-SD module, forcibly working to reduce the influence of energy loss with the aim of using the flow demand change (F_DC_) and stock of electricity demand (S_ED_). F_DC_ is a transition function for several parameters: (1) maximum and minimum demand (D_MM_), (2) price of elasticity demand (P_ED_), (3) baseload line demand (*L*) and (4) user fees (U_F_). The converter (C_ON_) of (D_MM_) is used to set a maximum/minimum electricity demand limit (See Fig. 10).

The index of P_ED_ is the percentage change in the demanded quantity of electricity divided by the corresponding percentage change in price [17]. The function of demand change (F_DC_) aims to reduce electricity demand from the grid by suggesting alternative utilities in a household if users’ fees increase based on a specific goal [17]. The rationale for the decrease in electricity demand is that electricity conservation measures are applied in households to reduce electricity costs that affect supply and demand, the optimum goal of average demand (*X*_*i*_). Thus, price-induced changes in electricity consumption will be avoided in peak/off-peak times. Therefore, V_CL_ in BBA assumes the total electricity consumption is the product of a specific time for the population served by the utility and per capita, where electricity demand displays the billable volume of electricity and earns revenue for the utility. When a large proportion of consumers' chain logic (V_CL_) is in poor condition, it incurs a significant loss of energy. The electricity capacity delivered into the distribution network will be higher than the total electricity consumption, where these extra costs are associated with operational energy expenditures.

**Fig. 11:**
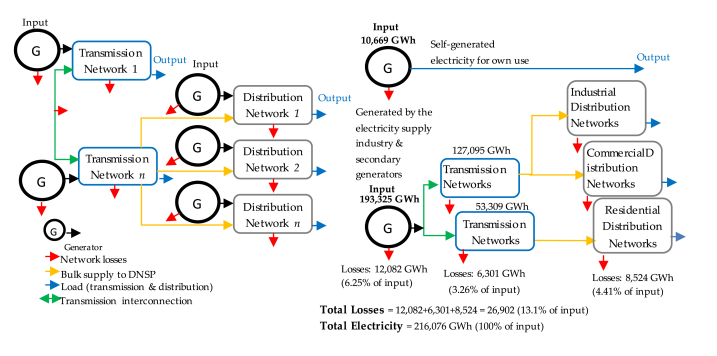
Supply chain Vs. energy loss of electricity [63].

Demand loads and generators for transmission and distribution are unequal, making the output of the transmission networks uneven as the bulk supplies to the distribution networks are connected to them [91]. The average loss occurring in generation transmission and distribution networks is respectively 6.25 %, 3.26 % and 4.41 %, as the combined average loss is estimated to make the total loss in Australian electricity networks 13.1 % of the total inputs (see Fig. 11). However, the energy loss in grid systems is still under investigation because energy loss resulting from unfixed and differently integrated factors makes it hard to consider the loss fixedly [71].

As we clearly can see, the energy loss occurs at each stage of the power grid system, beginning from power generation, passing through step-up transformers to connect the power supply to the transmission system, passing through the power distribution, and ending with the end-users wiring beyond the retail meter. All the associated losses, referred to as line losses, are mainly affected by the electricity demand in the highest peak hours, disproportionately more flow of electricity needed through the transmission conductors to cover end-users demand. In the same manner, off-peak demand hours are subject to losses due to the end-users demand being dropped down under the minimum generation capacity schema of safe operation, which also leads to energy losses. Therefore, V_CL_ is subject to providing particular tangible and intangible attention to this issue and an opportunity for efficiency improvement. As depicted in Table 3, V_CL_ shows and assesses the issue's significance and values without associating a solution; obviously, it is the role of V_NL_ and V_SL_.

**Table 3:** V_CL_ loss average results.

V_CL_ Energy Loss Results			
	Generation	Transmission	Distribution
*Error(%)*	6.25 %	3.26 %	4.41 %
*Accumulative Error(%)*	6.25 %	6.61 %	11.02 %
*Total Errors(%)*	11.02 %		

Some regulation Impact Statements have been formed to define efficient ways for network expansion. Those statements highlight that there is a lack of reliable and publicly available information on losses and trends occurring through transmission and distribution networks. Besides, network businesses and specialised agencies are not tasked with consistently and systematically gathering and reporting losses in information off the grid. This lack of transparency could be addressed as an addition to the reporting requirements through the electricity regulatory frameworks [92].

### 9.2 Black box B (BBB-V_SL_)

The self-sustaining financial utility of electricity retailers implies the ability to maintain a zero funds balance by investing the revenues in reducing the transition at peaks and off-peak demands equal to the total of capital and operational expenditures [17,93]. In designing an appropriate V_SL_ representation of solutions to energy loss, end-users demand is supposed to be stochastic, and the BBB module formulation, according to stationary cost parameters of a peak, off-peak and average overtime demand. *BBB* employs V_SL,_ in which two main aspects will be linked to stock flow and interconnected, and those are using electricity fees and structure funds balance (*R3*) (see Fig. 9). R3 illustrates the net funds at the end of each specified period and will be replenished through the revenue flow (B1) [17]. There are many ways to estimate the fees; however, a constant capacity of the user fees (U_F_) regime has been used in the analysis. The grid revenue is calculated according to the level of electricity used by end-users concurrent with the average consumption (*X*_*i*_) and the *U*_*F*_. Capital expenditure (CapEx) illustrates the rehabilitation costs to change a household from one ‘Stock Condition’ group to another, starting from the worst-case scenario of 100 % as the most unsatisfactory condition to 0 % as the final best-case scenario. Flow CapEx is defined by the electricity storage capacity and generation and the unit price of a house rehabilitation (CapEx ∗ UnitPrice) [17].

Operational expenditures (OpEx) display unaccounted electricity loss during changing grid plans, emergencies or maintenance needed in a household for energy-installed sources. U_F_ tracks the electricity price per the electricity charged to individual consumers. U_F_ allowed varying scenarios consequent to the demand level diverging from the baseload line. Equalising revenues and expenses, then Stock User Fees are controlled through outflow User Fee Decline (*U*_*FD*_) and Inflow User Fee Hike (U_FH_) [17]. Using this theoretical model to serve a household utility in short optimisation loops. Building this model attempts to find answers about what threats exist to a household scale's sustainability [[93], [94], [95], [96], [97], [98], [99], [100], [101], [102], [103], [104], [105], [106], [107]].

Taking the V_SL_ role in *BBB*, this time, we used the light Sensitivity analysis *(SA)* technique to investigate this unit of the IC_*T*_ module [14]. Sensitivity analysis *(SA)* was used based on three proposed patterns of electricity demand: *off-peak demand (l), peak demand (u) & average demand (AV);* against three main parameters of traditional and renewable power sources: *controlled load demand (CL), general consumption at home (GC) & solar panels at home (GG)*. The purpose of simulation analysis is to measure the values of IC_*T*_ based on the role of V_SL_ influenced by the integration of V_CL_ and V_NL_ units in mitigation of the effect of *(l)* and *(u)* and enhance the effect of *(AV)* (see Fig. 10). In consequence of this, deployment time series data of smart home meters have been manipulated to test IC_*T*_ for developing system performance.

(6) Minimise f(x)

(7) Subject to g_i_(x) = 0, i = 1, m

(8) $$X=\left(y,x\right),\bar{x}=\left(\bar{y},\bar{x}\right)$$

(9) *g (y, x)* = *0*

(10) *g* = *(g1, …., gm)*

(11) *f(x)* = *f(y(x), x)*

Reducing the problem to an average range within lower and upper bounds:

(12) *Normalise f(x)*

(13) *Subject to l*_*i*_ *≤* *X*_*i*_ *≤* *u*_*i*_*, i* = *1, n*

(14) *Assuming AOp* *≥* *POp implies a unique solution*

(15) $$Problem\;reduction\;starting\;from\;x_{0}\equiv \bar{x},and\;i=0$$

(16) *Testing the optimality of the new results of Xi* = *(y*_*i*_*, x*_*i*_*)*

(17) *H* = *∂*^*2*^*f* = *∂x*_*i*_*/∂x*_*j*_

(18) $$f\left(x+\Delta x\right)\approx f\left(x\right)+\sum_{i=1}^{n}\Delta x_{i}\frac{\partial f}{\partial x_{i}}+\frac{1}{2}\sum_{i=1}^{n}\Delta x_{i}{\Delta x}_{j}\frac{\partial^{2}f}{\partial x_{i}{\partial x}_{j}}$$

(19) *Control demand between lower (l*_*i*_*) and upper bounds (u*_*i*_*): l*_*i*_ = *AV*_*min*_*, u*_*i*_ = *AV*_*max*_

(20) $${{(L}_{ij}^{n})}_{c}={\left(GC+CL-GG\right)}_{POp}$$

(21) $${{(L}_{ij}^{n})}_{d}={\left(GC+CL-GG\right)}_{AOp}$$

(22) *(OG)*_*GC+CL-GG*_ = *(DCBO)*_*AOp*_*– (DCAO)*_*POp*_

**Fig. 12:**
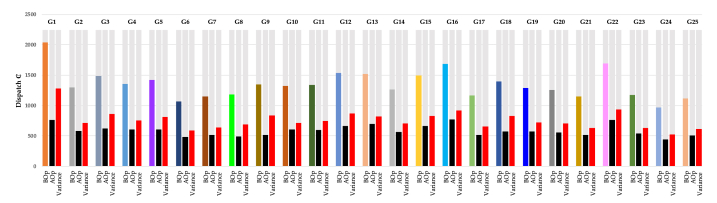
Clustered columns of optimisation results “variance, *BOP* and *AOP”*.

Three results values of IC_*T*_ will be achieved from the simulation optimisation analysis. The simulation goal is to keep the original optimisation set of (i = 1), subject to *l*_*i*_ *≤* *X*_*i*_ *≤* *u*_*i*_ where *l*_*i*_ and *u*_*i*_ are the minimum and maximum thresholds vectors for *i.* The purpose of simulation analysis is to measure the values of IC_*T*_ based on the role of V_CL_, V_SL_ and V_NL_ in mitigation of the effect of (l) and (u) and enhance the effect of (AV). SA techniques, such as GRG Nonlinear, LP/Quadratic, SOCP Barrier, Evolutionary and Intemodelal, can be used to model the electricity demand profiles. Time series data of smart home meters have been computed based on the concerns of IC_*T*_ for developing system performance. However, the model of time series data of smart home meters has been diagnosed as “LP Convex quadratic”, where all expected solutions are timely and linear combinations of current-based-decision variable values, which there is no one solution satisfying the constraints or a better value for the objective and it is expected there is a range of timely solutions with the same objective value. The solution has an expectation of multiple decision variable values as should emerge within combinations of linear solutions from different variables (end-users) in a timely order. The achieved result in Fig. 12 and Tabl4 has a Parse time of 14.09 Seconds, and an analytical solve time of 18.55 Seconds.

The SA analysis is computed and displayed in the bar chart in Fig. 12, and Table 4 to study the rates' impacts of energy loss of individual end-users based on the V_SL_ optimisation scenario to the home demand (GC + CL-GG). The simulation results display (SA) analysis's concern against the threats of energy loss to home sustainable systems. Estimation of the float of energy loss associated with a specific cost to end-users, it is observed that the average profit attains the maximum value of 1281.59 (currency unit) at *B*_*OP*_ = 2041.36 and a minimum value of $521.64 at *B*_*OP*_ = 966.01 when the demand rate crosses *X*_*i*_ desired unit per unit time, in both cases, the average profit goes up (See Table 4).

**Table 4:** Numerical values of optimisation results “variance, *BOP* and *AOP”*.

*Dispatch ₵*	**G1**	**G2**	**G3**	**G4**	**G5**	**G6**	**G7**	**G8**	**G9**	**G10**	**G11**	**G12**	**G13**	**G14**	**G15**	**G16**	**G17**	**G18**	**G19**	**G20**	**G21**	**G22**	**G23**	**G24**	**G25**
*BOp*	2041.4	1299.8	1485.4	1355.1	1419.3	1069.7	1152.9	1183.4	1346.1	1319.7	1340.2	1533.7	1517.4	1262.6	1492.4	1686.5	1167.3	1400.1	1293.7	1259.7	1147.9	1693.6	1172.8	966.0	1113.8
*AOp*	759.8	584.3	620.7	604.8	609.5	478.8	516.9	493.3	511.9	607.1	594.0	663.8	698.0	562.1	665.4	766.7	515.8	570.4	572.1	559.2	516.1	761.5	539.2	444.4	503.1
*Variance*	1281.6	715.6	864.8	750.3	809.8	590.8	636.0	690.1	834.2	712.7	746.2	869.9	819.4	700.5	827.0	919.8	651.5	829.7	721.6	700.4	631.7	932.1	633.6	521.6	610.7
*Error (%)*	62.8 %	55.1 %	58.2 %	55.4 %	57.1 %	55.2 %	55.2 %	58.3 %	62.0 %	54.0 %	55.7 %	56.7 %	54.0 %	55.5 %	55.4 %	54.5 %	55.8 %	59.3 %	55.8 %	55.6 %	55.0 %	55.0 %	54.0 %	54.0 %	54.8 %

**Fig. 13:**
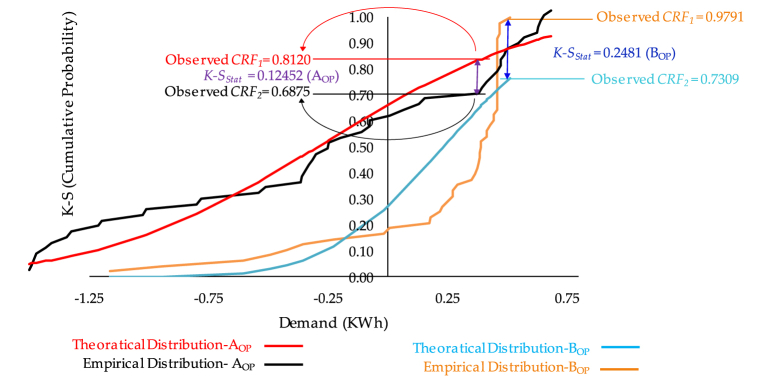
Kolmogorov-Smirnov (*k-s*).

Kolmogorov-Smirnov (*k-s*) of Piecewise linear distribution, used along with critical value (*cv*) and probability value (*pv*) to test the normality of the data set and measure the empirical distribution function agreement before and after the system's optimisation. The outcome displays the goodness of fit of continuous random variables by comparing the clustering of individual systems with their individual states before (*B*_*OP*_) and after (*A*_*OP*_) of data optimisation. At all cases, *k-s*_*stat*_ found increase constantly online with *pv* vlaues and always higher than *cv* values as deiced in Fig. 13 and Table 5.

**Table 5:** Comparison between *k-s* and *cv*.

***p-value (α)***	**0.001**	**0.01**	**0.02**	**0.05**	**0.1**	**0.15**	**0.2**
*k-S*_*stat*_*(B*_*OP*_*)*	0.12452	0.12452	0.12452	0.12452	0.12452	0.12452	0.12452
*C-value (B*_*OP*_*)*	0.12329	0.10293	0.09597	0.08589	0.0774	0.07197	0.06784
	*CV* *<* *k-S*	*CV* *<* *k-S*	*CV* *<* *k-S*	*CV* *<* *k-S*	*CV* *<* *k-S*	*CV* *<* *k-S*	*CV* *<* *k-S*
*k-S*_*stat*_*(A*_*OP*_*)*	0.24817	0.24817	0.24817	0.24817	0.24817	0.24817	0.24817
*C-value (A*_*OP*_*)*	0.12329	0.10293	0.09597	0.08589	0.0774	0.07197	0.06784
	*CV* *<* *k-S*	*CV* *<* *k-S*	*CV* *<* *k-S*	*CV* *<* *k-S*	*CV* *<* *k-S*	*CV* *<* *k-S*	*CV* *<* *k-S*

It is vital that we have noted the spatiotemporal impact that V_SL_ is having on a reduction in various ranges of energy loss ($L_{\text{ij}}^{n}$). It is also noted that the subproblem solution of V_SL_ is feasible for all maximum and minimum demand rates, and the number of errors when fixing it reduces the loss and converts it to profit, which can be concluded in Table 6.

**Table 6:** V_SL_ optimisation average results.

V_SL_ Optimisation Average Results			
	Max.	Mean	Min.
Error (%)	62.78		54.00
No Error (%)	37.22		46.00
Annual expected profit $ per End-users	1281.59		521.64

This result takes to a V_SL_ contribution to the grid system. Nevertheless, we must not use the way V_SL_ contributes to this study as methodical compliance to provide a fixed role in running a system with quantitative information in making decisions. Because the origin of its ‘base’ has no link to one fixed solution idea, the analysis idea is born out of the goal we want to achieve and is specifically designed for this study. What we are left with, basically, is that the extension of V_SL_ maintains solutions based on the experience and observation of individual 'attitudes' regarding how we perceive them.

### 9.3 Black box C (BBC)

End-users use the utility of electricity service through multiple contractual tariff schema (time of using tariffs, capacity & demand-based tariffs and use of non-utility-based communications tariffs). Concurrent with the influence of energy loss during peak and off-peak times [108]. *BBC* unit proposes multiple modes of connections inspired by V_NL_ to influence consumers' behaviours. By returning to Fig. 11, ‘Stock home condition’ (S_HC_) displays the need for rehabilitation to change a household from one ‘Stock Condition’ group to another. 100 % as a most unsatisfactory condition through descending steps of improving stock conditions 100, 80, 60, 40, 20 and 0 %, which zero meaning the optimum best condition state. With a balancing penetration of renewable energies in individual households, electrical individual end-user systems increase to provide valuable services to the grid versus energy loss.

Based on analogies between energy loss management and the performance of homes' electrical energy systems, the V_NL_ analysis is the pioneer attempt to adopt suitable individual end-user systems under the perception of the HAN coordination problem. An ideal load profile proposed in this study as a horizontal line within the maximum and minimum ranges of *X*_*i*_ power demand. The occurrence of energy loss is estimated based on the variety of end-user demand, i.e., *l*_*i*_, *u*_*i*_, *X*_*i*_. This issue is escalating to more complex levels at residential houses where all physical limitations of the power generation, transmission and distribution are accumulated and rated.

The role of V_NL_ is quite critical, depending on the feedback information from the V_CL_ and V_SL_ to demonstrate how much renewable energy is needed at home to flatten peaks and off-peaks to the range of *X*_*i*_ demand. V_CL_ and V_SL_ are not provisioned for the fluctuation of power demand. With illustrations of the literature review, modelling and simulation outputs listed in Table 1, Table 2, Table 3, Table 4, Table 5, and Fig. 6, Fig. 7, Fig. 8, Fig. 9, Fig. 10, Fig. 11, Fig. 12, originally, the objective functions are set to the purpose of V_CL_ and V_SL_. And at this stage, to demonstrate V_NL_ role, we have used a generalised reduced gradient algorithm (*GRG*) to detect the nonlinear optimisation issue caused by end-user demand that yields a better value for the objective. With *GRG*, although there is a range of different values for the variables but can yield better values for the objective to satisfy the constraints. Therefore, f(x) = f(y(x), x) and (H = ∂2 f = ∂xi/∂xj) of Taylor expansion was applied to limit the occurrence of ($L_{ij}^{n}$) in turn, thus controlling the cyclic demands to the objective (*X*_*i*_) that we have selected to study. In the *V*_*NL*_ model optimisation, we compute individual systems with maximum charging and discharging rates of storage battery devices to support individual end-users home systems against energy loss. If the capacity is needed of BCh > BDi then there is no need for solar capacity to serve the battery in the daytime to cover the lack of capacity at night time.

(23) $$B_{Ch}=f\left(\sum_{t_{d}}H_{dl}*t-\sum_{t_{d}}X_{i\left(\min\right)}*t\right)$$

(24) $$B_{Di}=f\left(\sum_{t_{n}}X_{i\left(\max\right)}*t-\sum_{t_{n}}H_{dl}*t\right)$$

(25) $$B_{TC}=f\left(\sum_{t_{d}}H_{dl}*t-\sum_{t_{d}}X_{i\left(\min\right)}*t\right)+f\left(\sum_{t_{n}}X_{i\left(\max\right)}*t-\sum_{t_{n}}H_{dl}*t\right)\;\forall B_{C}$$

(26) $$B_{Ch}+B_{Di}\leq 1\;\forall t$$

The strategy of this optimisation defines the optimal capacity of storage battery and *P*_*V*_ recommended to individual end-users to make up its deficit and control its surplus based on the objective function *X*_*i*_ that we have discussed via the previous sections of *V*_*CL*_ and *V*_*SL*_. To formulate the strategic objective in this study, Network value represent the individual systems of end-users where *n* **∈** *X*_*i*_ at *t,* where *X*_*i*_ **∈** *S*_*DT*_ and *S*_*NT*_ *at t* which can be given as:

(27) $$S_{DT}=f\left(\sum_{t_{d}}H_{dl}*t-\sum_{t_{d}}X_{i\left(\max\right)}*t\right)$$

(28) $$S_{NT}=f\left(\sum_{t_{n}}H_{dl}*t-\sum_{t_{n}}X_{i\left(\max\right)}*t\right)$$

where *S*_*DT*_ and *S*_*NT*_ are the maximum predicted capacity needed of PV power generation to serve the home during daylight (directly) and night-time (via storage batteries) respectively at interval time *t.* The constraints *P*_*V*_*2G, P*_*V*_*2L* and *P*_*V*_*2B* are the amount of energy that solar power provide to the grid, demand load and storage battery subsequently and given as:

(29) $$S_{TC}=P_{t}^{P_{V}2L}+P_{t}^{P_{V}2G}+P_{t}^{P_{V}2B}\forall t$$

Finally, the *S*_*TC*_ in (30) denotes to the total PV capacity required for individual homes' optimisation, the upper demand thresholds during daylight H_*dl*_ *(t*_*d*_*)* and nighttime H_*dl*_ *(t*_*n*_*),* and the maximum of average demand *X*_*i(max)*_ maintain the total amount of energy expected to generate from the *P*_*V*_ power at individual homes. We have enforced the module with a boundary condition by assuming that the total solar capacity *S*_*TC*_ and the total battery capacity *B*_*TC*_ determine the non-variable optimisation *n* to the load *L* during 0.5 unit of interval time *i* to the period *J* must equal to the objective of desired average demand *X*_*i*_ as formulated in (31).

(30) $$S_{TC}=f\left(\sum_{t_{d}}H_{dl}*t-\sum_{t_{d}}X_{i\left(\max\right)}*t\right)+f\left(\sum_{t_{n}}H_{dl}*t-\sum_{t_{n}}X_{i\left(\max\right)}*t\right)\;\forall S_{C}$$

(31) $$L_{ij}^{n}=f\left(S_{TC}\right)-X_{i}+B_{TC}\;\forall L_{C}$$

We use the conventional grid power model *P* where the grid supply unit *G*_*P*_ at time *t* denoted for two purposes; *G*_*P*_*2L* supplies power to the end-users demand *GC* and *CL*, while *G*_*P*_*2B* delivers the power from the grid to charges the storage batteries *B*_*Ch*_ at homes based on individual module strategies of end-users, and can be formulated as:

(32) $$P_{t}^{G_{P}}=P_{t}^{G_{P}2L}+P_{t}^{G_{P}2B_{Ch}}$$

The objective of this study is that the grid power *G*_*P*_ at time *t* should satisfy surplus and deficit demand constraints denoted to off-peak *li* and peak *u*_*i*_ demands and as presented in (33).

(33) $$l_{i}\leq P_{t}^{G_{P}}\leq u_{i}$$

*L (t)* is a monocular and strictly constraint function due to the independency of end-users demand systems and their interdependency on aggregate load at each *t* ∈ *j, i, n*. Dynamic demand parameters of end-users given in (38) and defined as $L_{n}^{ij}=GC+CL\pm GG$ at *t*. It is also the amount of grid energy rate capability identified before we simulate end-users systems. A linear combination of *L* at *t* within the ranges of desired demands *X*_*i(min)*_ and *X*_*i(max)*_ given in (34) and (36) is applied to reduce the influence of the continuity constraints that emerged from different end-users demands as given in (35) and (37).

(34) $$X_{i\left(\min\right)}\leq L_{n}^{ij}\leq X_{i\left(\max\right)}\forall t$$

(35) $$L_{n}^{ij}=GC+CL\pm GG\;\forall t$$

(36) $$Binary\;variable:=1\;when\;i\;goes\;l\_\left(i\right)\leq GC+CL\pm GG\leq u\_\left(i\right),\left(optimum\;demand:L_{ij}^{N}=x_{i}\right)\;\forall t$$

(37) $$Binary\;variable:=2\;when\;i\;goes\;u\_\left(i\right)\leq GC+CL\pm GG\leq l\_\left(i\right)\;\left(none\;optimum\;demand:L_{ij}^{N}\neq x_{i}\right)\;\forall t$$

This section of V_NL_ model investigates the logic of renewable energy capacity at individual homes. The algorithm proposes that every end-user has an accurate prediction of the network asset of renewable energy to avoid non optimum demand when $L_{ij}^{N}\neq x_{i}$. The sustainability of end-users' systems presented in the simulation results in Table 7 adopt an original intellectual capital thinking technique of V_NL_ applied via a system dynamic framework by involving multi-users demand scenario.

**Table 7:** V_NL_ optimisation average results.

HAN	*P*_*V*_ Per Home (KWh)		*B*_*∏*_ Per Home (KWh)			G_P_ (KWh)	
Unit (KWh)	Per End-user	Per Year	Charging	Discharging	Per End-user	Per End-user	Per Year
End-users	250	250	250	250	250	250	250
Mean	1.71	622.46	0.14	0.44	0.58	0.63	221.64
Min.	1	365	0	0	0	0.49	4297.16
Max.	9.99	3646.35	7.27	22.03	29.3	0.78	6789.93
Highest Capacity	2.41	881.23	19.15	18.67	37.82	1.58	578.48
Lowest Capacity	0	0	6.27	14.1	20.37	0.19	68.64
Average Capacity	0.66	242.48	12.57	17.2	29.77	0.64	232.31
Available Capacity	426.34 (71.97 %)	155614	0	0	0 (0 %)	5587.59 (100 %)	1396897.5
Required Capacity	166.08 (28.03 %)	60619.2	314.323 (42.21 %)	430.01 (57.78 %)	744.34	0.00 (0.00 %)	0
Total G_P_, P_V_, B_TC_	592.42 (100 %)	216233	744.34 (100 %)		744.34 (100 %)	5587.59 (100 %)	1396897.5
Total STC (19.29 %)	44.30 %				55.68 %		
Total Per Year (89.26 %)	9.58 %					90.42 %	
HAN Capacity (100 %)	8.55 %				10.74 %	80.71 %	

By considering the original demand of discrete time record *L(t)* and demand starting time *t* = *t*_*0*_; numerically evaluated *S*_*TC*_ for the formula number (32), which denoted to the predictor *H* ∈ *l*_*i*_, *u*_*i*_ and *X*_*i*_ (see Table 6). To make it simple, within the mean of discrete series demand data $L_{ij}^{N}=x_{i}$ in this simulation, we have assumed the simulation optimisation of *P*_*V*_ and storage battery capacities *B*_*TC*_ those required to the system *S*_*TC*_ are the 100 % capacity needed and no inefficiencies of storage batteries or conversion or leakage cause rated losses which that will take us to other research venues might need to be handled separately in other related studies.

The optimisation results of alternative variables of *P*_*V2*_ correspond to the capacity available of *P*_*V1*_ of the basic variables in the original time series data. In a genuine deterministic flow, one can see the capacity required of *P*_*V2*_ ∈ *AO*_*P*_ (after optimisation) to all end-users found to be 28.03 % in addition to 71.97 % of *P*_*V1*_ ∈ *BO*_*P*_ (before optimisation) randomly distributed and available chaotically at homes systems. The numerical computation of *S*_*TC*_ at *t*, displays 8.55 % is the optimisation result of the capacity required that compulsory should be added to end-users systems to obtain the total capacity that is able to achieve the objective *X*_*i*_.

At this point of the simulation, we are left to determine *B*_*TC*_ as the second renewable energy resource needed at home. The analysis results are in Table 6; we shall observe that this study's manipulated end-user sample has no storage battery values installed at homes. The primary factor in calculating the *B*_*TC*_ in this study is the time slot where an action can be captured depending on the end-user demand. In our case *L* ∈ *l*_*i*_ and *u*_*i*_, the capacity of charging *B*_*Ch*_ and the capacity of discharging *B*_*Di*_ are bounded according to the constraint $l_{i}\leq P_{t}^{G_{P}}\leq u_{i}$ where $P_{t}^{G_{P}}$ ∈ *X*_*i*_ devoted to the optimisation objective adopted for the analysis. *B*_*TC*_ balance between the consumed and available electricity in each time slot. We simulate errors in predicting the total time of battery device usage where 314.32 charging units (42.21 %) of *B*_*Ch*_ and 430.01 discharging units (57.78 %) of *B*_*Di*_ are denoted to the total forecasting capacity of *B*_*TC*_.

## 10 Limitation

A limitation of this study is that it focuses on the demand side management (*DSM*), on the segment of residential households in the electrical smart grid systems. The attempt we made to combine information from an intangible managerial perspective in a way that bounds on the strengths of tangible system assets to parametrise how these resources manage ‘Sustainable Human Future’. To clarify where this study was heading, we emphasised the intellectual capital thinking (IC_*T*_) functional role in praising three intangible logic values named value chain logic V_CL_, value shop logic V_SL_ and value network logic V_NL_, where the expected benefit of the IC_*T*_ approach pays particular attention to unforeseen intangible resources to improve system performance.

## 11 Conclusion

The main contribution of this study is summarised in the following points: (1) Assessing the impact of an intangible asset along with the tangible asset on the energy loss-driven cost by end-users, (2) Qualitative and quantitative analysis models are formulated based on a case study on demand data of end-users, (3) The possibility of adding root causes allocation approaches upon the issue of energy loss are considered. The *BBA* representation of *V*_*CL*_ illustrates the cumulative energy loss occurrence across generation transmission and distribution networks, estimating a total loss in Australian electricity networks approximately 13.1 % with a potential error rate up to 11.02 %. Meanwhile, V_SL_ depiction in *BBB* maintains views upon individual demand rates reflecting purchasing behaviour, revealing the energy loss rates at end-users demand level ranging from 62.78 % to 54 %. In *BBC*, the V_NL_ representation proposes multiple connections influenced by consumers' demand behaviours. It concludes that 42.21 % of charging units and 57.78 % of discharging units contribute to the overall forecasting capacity of *BTC*. Briefly revisit the optimisation results to showcase the total home and grid power required capacities for GP, S_TC_, and *B*_*TC*_. It can be seen in Table 7, the capacity of 80.72 % goes to the conventional power *G*_*P*_, 10.74 % is the total capacity of storage batteries recommended to install at homes, and 8.55 % is the total *P*_*V*_ capacity. The optimisation results show that we have 19.43 % of renewable energy from solar and storage batteries and 80.71 % of conventional energy from the grid generators. The particular illustration of *V*_*NL*_ demonstrates how shifting power usage can command and control energy demand by using different perspectives and techniques. The significance of the intellectual capital thinking of *V*_*NL*_ gives the sense to encompass grid-connected microgrid systems where end-users are interconnected with renewable energy resources from individual end-user networks. In formulating the suitability of the *V*_*NL*_ framework of energy demand management, we found each end-user demand network is designed with the optimal amount of renewable power storage and generation across the periods from *t*_*0*_ to *t*_*n*_. This period is sufficient to share and break through conventional grid power to cover the maximum demand of end-users and reduce energy loss.

This study speculates Intellectual Capital Thinking (IC_*T*_) to closely monitor and share the case of grid demand based on different frameworks of logical thinking values defined as V_CL_, V_SL_ and V_NL_. In a position mirrored by assessing exclusively three intangible values that align with the goal of this study, we have brought up in the abstract and concluded them as follows:

• Value Chain Logic (V_CL_) devotes functional management to representing energy loss found driven by 11.02 % total error in electricity resources based on several unforeseen formats displayed in Table 3.
• The simulation results displayed by sensitivity analysis (SA) concern the value shop logic (V_SL_) regarding the threats of energy loss to sustainable home systems. Three parameters were found to influence V_SL_: (1) General consumption demand (GC), (2) Controlled load demand (CL) and (3) solar generation (GC). Based on the V_SL_ optimisation average results in Table 4, the error range caused by V_SL_ found significance between 62.78 % and 54.0 %.
• At the same time, Value Network Logic (V_NL_) is meant to plunge human capital resources into advanced forms, converting them from intangible to dispatchable alongside traditional tangible resources. V_NL_ investigates the logic of renewable energy capacity of solar and storage batteries required at individual homes, which found that 28.03 % is needed in addition to 71.97 % in the current system. The numerical computation of storage capacity (STC) displays 8.55 % as the optimisation result of the capacity required to be added to end-users individual systems to obtain the total capacity to achieve the objective *Xi.*

This study shows the rallying goal of IC_*T*_ behaves utterly differently from other tangible strategic goals. The three IC_*T*_ logical values correspond logically to the false and true semantics, recommending specific algorithmic approaches for designing micro-level sustainable systems. The outcomes of this study demonstrated that IC_*T*_'s contribution to energy loss could improve the performance of a grid system. In particular, the value logic targeted in this study ought to support the efficiency of electric smart grid systems in some manner, besides thoroughly considering optimal system design to realise the level of variance estimation problems and how they can be detected and dealt with. In this study, there appear several answers to what can be done to stop energy loss, and the only settlement we made is that we want to know how far IC_*T*_ could advance a system from the inside context. This qualitative and quantitatively intangible study grasps theoretical and practical intangible values and shows the possible links with an organisation. The proposed IC_*T*_ of a ‘home sustainable module’ comprise factors suited to the study of a sustainable approach. Importantly, the three value logic methods of IC_*T*_ provide measures of how much each varying parameter contributes to the overall variance of the module performance. At the same time, the proposed parameters, constraints and variables of IC_*T*_ contribute to how the grid system operates.

## CRediT authorship contribution statement

**Ashraf Zaghwan:** Writing – original draft, Visualization, Software, Resources, Project administration, Methodology, Investigation, Formal analysis, Data curation, Conceptualization. **Yousef Amer:** Writing – review & editing, Investigation, Conceptualization. **Mahmoud Efatmaneshnik:** Writing – review & editing. **Indra Gunawan:** Writing – review & editing, Validation, Supervision.

## Data availability statement

The data underpinning the outcomes of this study will be accessible upon request. Meantime, supplementary datasets can be found in the Australian Energy Market Operator (AEMO) repository, adhering to AEMO's usability rules and standards and with their permission.

## Declaration of competing interest

The authors declare that they have no known competing financial interests or personal relationships that could have appeared to influence the work reported in this paper.
